# A Green Microbial Fuel Cell-Based Biosensor for In Situ Chromium (VI) Measurement in Electroplating Wastewater

**DOI:** 10.3390/s17112461

**Published:** 2017-10-27

**Authors:** Li-Chun Wu, Teh-Hua Tsai, Man-Hai Liu, Jui-Ling Kuo, Yung-Chu Chang, Ying-Chien Chung

**Affiliations:** 1 Department of Logistics Engineering, Dongguan Polytechnic, Dongguan 523808, Guangdong, China; mic.wu@msa.hinet.net; 2 Department of Chemical Engineering and Biotechnology, National Taipei University of Technology, Taipei 10608, Taiwan; thtsai@ntut.edu.tw; 3 Department of Food Science, China University of Science and Technology, Taipei 11581, Taiwan; manhailiu@cc.cust.edu.tw; 4 Department of Biological Science and Technology, China University of Science and Technology, Taipei 11581, Taiwan; iamlovewish@gmail.com (J.-L.K.); yji3w6@gmail.com (Y.-C.C.)

**Keywords:** chromium, biosensor, microbial fuel cell, wastewater, green production

## Abstract

The extensive use of Cr(VI) in many industries and the disposal of Cr(VI)-containing wastes have resulted in Cr(VI)-induced environmental contamination. Cr(VI) compounds are associated with increased cancer risks; hence, the detection of toxic Cr(VI) compounds is crucial. Various methods have been developed for Cr(VI) measurement, but they are often conducted offsite and cannot provide real-time toxicity monitoring. A microbial fuel cell (MFC) is an eco-friendly and self-sustaining device that has great potential as a biosensor for in situ Cr(VI) measurement, especially for wastewater generated from different electroplating units. In this study, *Exiguobacterium aestuarii* YC211, a facultatively anaerobic, Cr(VI)-reducing, salt-tolerant, and exoelectrogenic bacterium, was isolated and inoculated into an MFC to evaluate its feasibility as a Cr(VI) biosensor. The Cr(VI) removal efficiency of *E. aestuarii* YC211 was not affected by the surrounding environment (pH 5–9, 20–35 °C, coexisting ions, and salinity of 0–15 g/L). The maximum power density of the MFC biosensor was 98.3 ± 1.5 mW/m^2^ at 1500 Ω. A good linear relationship (*r*^2^ = 0.997) was observed between the Cr(VI) concentration (2.5–60 mg/L) and the voltage output. The developed MFC biosensor is a simple device that can accurately measure Cr(VI) concentrations in the actual electroplating wastewater that is generated from different electroplating units within 30 min with low deviations (−6.1% to 2.2%). After treating the actual electroplating wastewater with the MFC, the predominant family in the biofilm was found to be *Bacillaceae* (95.3%) and was further identified as the originally inoculated *E. aestuarii* YC211 by next generation sequencing (NGS). Thus, the MFC biosensor can measure Cr(VI) concentrations in situ in the effluents from different electroplating units, and it can potentially help in preventing the violation of effluent regulations.

## 1. Introduction

The extensive use of chromium (Cr) in many industries (e.g., electroplating, steel production, and leather tanning) [1] and the disposal of Cr-containing wastes over large areas of land have resulted in the release of chromium-containing effluents into the environment [2,3]. Chromium, a metal, is present in the environment in two major stable oxidation states—Cr(VI) and Cr(III) [1]. Cr(VI) compounds exist mainly as chromates and dichromates [4]. They are highly soluble and mobile and are considered as acutely toxic because they can readily cross cell membranes via the sulfate anion transport system when the ambient pH exceeds 6 [3,5]. Ramirez-Diaz et al. (2008) suggested that Cr(VI) compounds are associated with several diseases, such as allergies, contact dermatitis, and lung cancer [6]. Hence, the detection of toxic Cr(VI) compounds is of great importance in order to maintain the quality and safety of our environment.

Several chemical analysis methods, such as ion chromatography, atomic absorption spectrometry, inductively coupled plasma mass spectroscopy, and colorimetric methods based on diphenylcarbazide, are currently being used for the detection of chromium in water samples [7,8]. These methodologies exhibit a high sensitivity and selectivity but involve expensive equipment, require additional chemical compounds, specialized training, and long measurement times [2,4]. Apart from these classical methods, several biosensors have also been developed for Cr(VI) measurement. Examples include amperometric enzyme-based sensors using cytochrome c3 or urease and cell-based sensors using bacteria or V79 cells [1,9,10,11]. They are attractive alternatives to the classical methods due to their less complex instrumentation and shorter measurement period times [4]; however, these methods are often limited to measuring low Cr(VI) concentrations (μg/L); thus, their dynamic ranges restrict their use to diluted samples only [7]. These biosensors need external power sources and/or additional probes (e.g., pH, dissolved oxygen, and electrical conductivity) to measure the changes in the concentration of Cr(VI), which is another unfavorable factor [12]. Moreover, most of the analytical methods/techniques for Cr(VI) concentration monitoring are conducted offsite and thus do not reflect real-time values.

A microbial fuel cell (MFC) is a self-sustaining device without the need for any power supply [13]. MFCs have been employed to convert wastewater to electricity by electrogenic bacteria [14]. Previously, the development of MFC was mainly targeted towards power generation; however, in recent times, the potential of MFCs as biosensors for the measurement of parameters such as biological oxygen demand (BOD), chemical oxygen demand (COD), dissolved oxygen (DO), volatile fatty acids (VFA), and toxins in wastewater, has been demonstrated [12,13,15,16,17,18]. As a biosensor, a MFC possesses several unique characteristics [19]. First, the power required for the MFC to function is obtained from the wastewater itself without the need for an external supply. Secondly, because MFCs are based on the microorganisms and not enzymes or animal cells, it simplifies the setup of the biosensor and prolongs its lifetime. Third, the voltage output of an MFC is directly dependent on the metabolic activities of the anaerobic electrogenic bacteria, indicating that MFC is considered to be a promising device to monitor the Cr(VI) concentrations in wastewater, if the appropriate bacteria are used.

Many microorganisms capable of reducing Cr(VI) under anaerobic conditions have been reported. These strains include *Pseudomonas dechromaticans*, *P. chromatophilia*, *Aeromonas dechromatica*, *Desulfovibrio desulfuricans*, *D. vulgaris*, *Geobacter metallireducens*, *Shewanella putrefaciens*, *S. oneidensis*, *Pantoea agglomerans*, *Agrobacterium radiobacter*, *Thermoanaerobacter ethanolicus*, *Pyrobaculum islandicum*, and *Exiguobacterium aurantiacum* [20,21]. These bacteria exhibit potentials for in situ or ex situ measurements Cr(VI) when used in an MFC.

The use of Cr(VI) as an electron acceptor in the cathode of an MFC has been demonstrated in several studies [22]. In these MFCs, Cr(VI) reduction at the cathode and electricity production were accomplished simultaneously; thus, the MFC is a power generation device [22]. Xu et al. (2015) developed a flat membrane-based MFC sensor to monitor the toxicity of wastewater containing Cr^6+^ but the relationship between the Cr^6+^ concentration and voltage output was not fully established [12]. Liu et al. (2014) used Cr(VI) as an electron acceptor in the anode of an MFC to develop a cube MFC sensor for monitoring Cr^6+^ shock (<10 mg/L) [19]. Similarity, Chung et al. (2016) inoculated *Ochrobactrum anthropi* YC152 in the anode of an MFC as an early warning device for excess Cr(VI) concentrations (<5 mg/L) [7]. These results suggest that the inoculums in the MFCs just tolerated Cr(VI) toxicity but did not remove Cr(VI). Most regulatory agencies prescribe that the maximum allowable level of Cr(VI) in an effluent is 0.5 mg/L [21]; however, the concentrations of Cr(VI) emitted during the processes should be strictly controlled or monitored for sustainable, clean, and green production. Thus, an MFC-based biosensor should be developed for the in situ measurement of a wide range of Cr(VI) concentrations. Because the wastewater produced during electroplating contains large amounts of chromium as well as electrolytes, isolation of the appropriate bacterial strain is required for application in an MFC. In this study, *Exiguobacterium aestuarii* YC211, a facultatively anaerobic, Cr(VI)-reducing, salt-tolerant, and exoelectrogenic bacterium, was isolated from the electroplating wastewater. It was inoculated in the anode of an MFC to evaluate its feasibility as a biosensor for in situ Cr(VI) measurement. Crucial operating parameters were established to optimize the performance of the MFC. The relationship between the voltage output and Cr(VI) concentration was investigated. Cr(VI) concentrations in artificial and actual wastewater were measured using the developed MFC-based biosensor and standard colorimetric methods to illustrate the accuracy of the self-sustaining device.

## 2. Materials and Methods

### 2.1. Bacterial Strains, Cultivation, and Identification

Soil and sludge were collected from the vicinity of an electroplating wastewater treatment plant in Sanchong, New Taipei City, Taiwan. The electroplating wastewater treatment plant experienced repeated Cr(VI)-pollution crises in 2016. The collected samples were centrifuged at 9000× *g* for 30 min and the obtained precipitates were inoculated in a 3 L working volume in a chemostat. Tryptic soy broth (TSB), supplemented with Na_2_Cr_2_O_7_, called TSBCr medium, was continuously fed to the chemostat. The TSBCr medium containing 20–150 mg/L of Cr(VI) was progressively added into the chemostat to acclimate the Cr(VI)-resistant or -reducing bacteria under the anaerobic conditions at a liquid retention time (LRT) of 16 h. After a 45-day acclimation period, several dominant strains were isolated from the chemostat solution containing 150 mg/L Cr(VI) by the spread plate method. Among these dominant strains, YC211 bacterium with the biggest colony size on the agar was selected for subsequent experiments.

To identify the isolated YC211 bacterium, the YC211 cells were lysed and subjected to DNA extraction. 16S rRNA gene amplification and sequencing were performed, as described previously [23]. The 16S rRNA gene sequence of the YC211 bacterium was compared using BLASTN programs to search for the nucleotide sequences in the NCBI website. The phylogenetic tree was constructed using a bootstrap neighbor-joining program, Clustal X (Ver. 2.0).

### 2.2. Bacterial Growth and Cr(VI) Removal

To obtain the growth curve of the YC211 bacterium, it was cultured in 300 mL of the TSBCr medium with 60 mg/L of Cr(VI) and incubated at 30 °C at pH 7.0 under aerobic and anaerobic conditions, respectively. Bacterial growth and Cr(VI) removal were monitored during the culture period. Simultaneously, the bacterial growth was measured at a wavelength of 600 nm using a UV-Vis spectrophotometer (Thermo Fisher Scientific Inc., Waltham, MA, USA) and by the spread plate method. Cr(VI) concentration was determined using a colorimetric method in this study.

### 2.3. Factors Affecting Cr(VI) Removal by the YC211 Strain

To determine the effect of different culture conditions on Cr(VI) removal by the YC211 bacterium, different kinds and concentrations (original to 1/10,000 dilution) of the broth (LB or TSB), different pH values (4–12), culture temperatures (15–45 °C), and NaCl concentrations (0–20%) were used in anaerobic conditions. In this study, the YC211 bacterium was first grown in a TSBCr medium containing 60 mg/L Cr(VI) at 30 °C for 22 h under anaerobic conditions and then inoculated in fresh broth to conduct subsequent experiments. The culture conditions were at 30 °C, pH 7, 1000X TSBCr medium (60 mg/L Cr(VI)), and 1.6 × 10^7^ cfu/mL of the YC211 bacterium, unless otherwise stated. The Cr(VI) removal efficiency and cell numbers were analyzed after 24 h cultivation periods. All of the experiments were conducted at least in triplicate in order to evaluate the accuracy and reproducibility of the obtained results.

### 2.4. Construction of the MFC-Based Biosensor and Its Operation

A dual-chambered MFC was constructed, as described in a previous report [7], with a little modification to work as the Cr(VI) biosensor. The MFC was comprised of an 8 cm × 8 cm × 8 cm acrylic cube. The anode and cathode compartments had working volumes of 170 mL (7 cm × 7 cm × 3.47 cm) each and the 2 compartments were physically separated using a proton exchange membrane (Nafion 117, DuPont Co., Fayetteville, NC, USA) with a surface area of 49 cm^2^. Graphite felt (18 cm^2^ surface area) was used for the electrodes and an OK line connected the electrodes through a variable resistor. TSBCr medium (500 mL, 60 mg/L Cr(VI)) containing 1.6 × 10^7^ cfu/mL of the YC211 bacterium was placed in a glass bottle and continuously recycled in the anode compartment of the MFC with a 500 Ω resistor using a submersible pump for cell enrichment or immobilization under anaerobic conditions at a 10-day LRT. The TSBCr medium and the anode compartment were kept anoxic by purging with nitrogen gas. The catholyte consisted of 100 mM phosphate-buffered saline (PBS) and 100 mM NaCl solution [13]. When the potential of the inoculated MFC reached a steady state, the biofilm in the anode was considered stable.

To understand the operating characteristics of the MFC biosensor, 1/1000 TSB (water sample I) and three other kinds of water samples were used as the anolytes. The circuit was adjusted using variable resistance (100–10,000 Ω) to evaluate the relationship between current density and power density. Water sample II contained 1/1000 TSB, 30 mg/L Cu^2+^, 25 mg/L Zn^2+^, 25 mg/L Ni^2+^, 10 mg/L Ca^2+^, and 10 mg/L Mg^2+^. Water sample III contained 1/1000 TSB and 20 mg/L SO_4_^2−^. Water sample IV contained 1/1000 TSB, 30 mg/L Cu^2+^, 25 mg/L Zn^2+^, 25 mg/L Ni^2+^, 10 mg/L Ca^2+^, 10 mg/L Mg^2+^, and 20 mg/L SO_4_^2−^. In these batch experiments, the voltage and power density of the MFC were analyzed after 60 min operating periods when the original anolyte in the MFC was completely replaced with the tested water samples. All of the experiments were conducted at least in triplicate in order to verify the accuracy and reproducibility of the obtained results.

After determining the operating external resistance on the basis of the relationship between the current density and the power density, 1 mL Cr(VI) with a final concentration in the range of 0.01–100 mg/L was added to 1/1000 TSB to analyze the relationship between voltage variation and reaction time. Based on these results, the relationship between the Cr(VI) concentration and voltage output of the MFC or the calibration curve (voltage vs. Cr(VI) concentration) was established.

### 2.5. Cr(VI) Measurement in Artificial and Actual Electroplating Wastewater

Cr(VI) concentrations in artificial and actual electroplating wastewater were measured using the developed MFC biosensor and a standard colorimetric method. As the measurable Cr(VI) concentration ranges are different for the MFC biosensor (2.5–60 mg/L) and the colorimetric method (0.1–1 mg/L), appropriate dilution of the water samples may be required. In this study, artificial wastewater contained the prepared Cr(VI) concentration (7.5–55 mg/L) in 1/1000 TSB solution. To evaluate the feasibility of the MFC biosensor, actual electroplating wastewater samples were collected. Wastewater samples A–D were obtained from the effluents of different electroplating units. To measure the Cr(VI) concentration in the wastewater, the original anolyte in the MFC was completely replaced with the artificial wastewater; however, in the case of the electroplating wastewater, 169 mL of actual wastewater was supplemented with 1 mL of 17/100 TSB to maintain the TSB concentration in the anolyte of the MFC. Then, Cr(VI) concentration in wastewater samples was directly determined by the MFC biosensor in batch mode. Based on the established calibration curve (described in Section 2.4), the Cr(VI) concentrations in these wastewater samples could be easily analyzed. All of the experiments were conducted using five separate MFCs and each analysis was conducted in triplicate.

### 2.6. Analysis

The standard colorimetric method for Cr(VI) measurement was performed as described previously [24]. Briefly, 200 μL of the water sample was initially made up to 1 mL with distilled water. Later, this solution was made up to 10 mL with 400 μL of 0.25% S-diphenyl carbazide, 330 μL of 6 M H_2_SO_4_, and distilled water. Subsequently, the Cr(VI) concentration in the solution was determined at 540 nm using a UV-Vis spectrophotometer (Thermo Fisher Scientific Inc., Waltham, MA, USA). The Cr(VI) removal efficiency of the YC211 bacterium was then calculated as follows:

(1) $$\mathrm{Cr}(\mathrm{VI})\;\mathrm{removal}\;\mathrm{efficiency}(\%)=\left(\frac{C_{i}-C_{f}}{C_{i}}\right)\times 100$$

where C_i_ and C_f_ are the initial and final Cr(VI) concentrations, respectively.

The potential difference between the anode and the cathode was measured using a Model 2700 multimeter (Keithley Instruments Inc., Solon, OH, USA). The data were continuously recorded on a computer using a Model PCI-488 interface card (Keithley Instruments Inc.). The current (*I*, *amp*) was calculated at a resistance (*R*, *ohm*) from the voltage (*V*, volt) as *I* = *V/R*. The power (*P*, *watt*) was calculated by *P = I × V*. The power density (mW/m^2^) and current density (mA/m^2^) were calculated by relating the power and current with the surface area (m^2^) of the anode, respectively. All of the analyses were conducted in triplicate and the mean values were calculated.

To understand the changes in the bacterial community of the MFC, the biofilm at the anode (i.e., the graphite felt) was collected for bacterial community analysis using NGS before and after determining the Cr(VI) concentration in the electroplating wastewater. Cell lysis, DNA extraction, polymerase chain reaction (PCR) amplification, and 454 pyrosequencing were conducted per the processes described by Naz et al. (2016) [25]. DNA was extracted using a Fast DNA SPIN Kit (MP Biomedicals). PCR primers GAGTTTGATCNTGGCTCAG (forward) and GTNTTACNGCGGCKGCTG (reverse) were used to amplify the eubacterial 16S ribosomal RNA fragment. PCR profiling was conducted as follows: 95 °C for 10 min, 35 cycles at 94 °C for 45 s, 55 °C for 1 min, 72 °C for 1 min, and a final extension at 72 °C for 10 min. All of the partial 16S rRNA gene sequences were preprocessed per the methods described by Naz et al. (2016) [25]. Sequence analysis was performed using the Quantitative Insights Into Microbial Ecology software package. These processed sequences were clustered into Operational Taxonomic Units (OTUs) based on 0.97 sequence similarity with the UCLUST algorithm. Representative OTUs were selected based on the most abundant sequences and taxonomic assignment was conducted using the ribosomal database project classifier.

## 3. Results and Discussion

### 3.1. Identification and Characterization of the Cr(VI)-Reducing Bacterium

The YC211 bacterial strain was Gram-positive, facultatively anaerobic, and cocci-shaped in stationary growth phases. The bacterial colonies were white, round, raised, glossy, and 3.5–4 mm in diameter after incubation for two days on TSA (tryptone soya agar) at 30 °C under anaerobic conditions. Growth occurred at 15 °C and 45 °C, with optimum growth in the 25–35 °C temperature range. The optimal pH range for the growth of the strain YC211 was 5–8. The YC211 bacterium was salt-tolerant and grew well in 15% NaCl. These characteristics of the YC211 strain were estimated using cell numbers.

**Figure 1:**
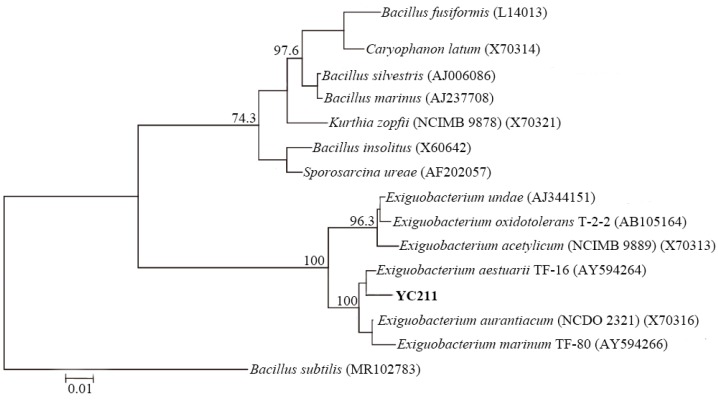
Neighbor-joining tree based on 16S rRNA gene sequences showing the phylogenetic relationship between YC211 strain and some other related taxa. The sequence of *Bacillus subtilis* (MR102783) was used as the outgroup. Bootstrap values greater than 50% are shown at nodes. Bar, 0.01 substitutions per nucleotide position.

The PCR amplification and sequencing procedures suggested by Corby-Harris et al. (2014) [23] were employed and resulted in the isolate being identified as *E. aestuarii* YC211. The results of the phylogenic analysis are presented in Figure 1. The confidence limit for each strain, as determined by the bootstrap method with 1000 resamplings, was >50% of the nodes. Theses bacterial sequences fell into two clades and one outgroup. Clade 1 was comprised of the sequences of *Bacillus fusiformis*, *Caryophanon latum*, *B. silvestris*, *B. marinus*, *Kurthia zopfii*, *B. insolitus*, and *Sporosarcina ureae*. Clade 2 was comprised of *Exiguobacterium* sequences. The phylogenetic tree analysis of the isolate indicated that the isolate YC211 had 98.7% similarity to *E. aestuarii* TF-16; both the YC211 isolate and *E. aestuarii* TF-16 were in the same cluster. According to the physiological study of *E. aestuarii* by Kim et al. (2005) [26], the YC211 bacterium had similar characteristics, which further demonstrated that the YC211 bacterium belonged to *E. aestuarii*.

**Figure 2:**
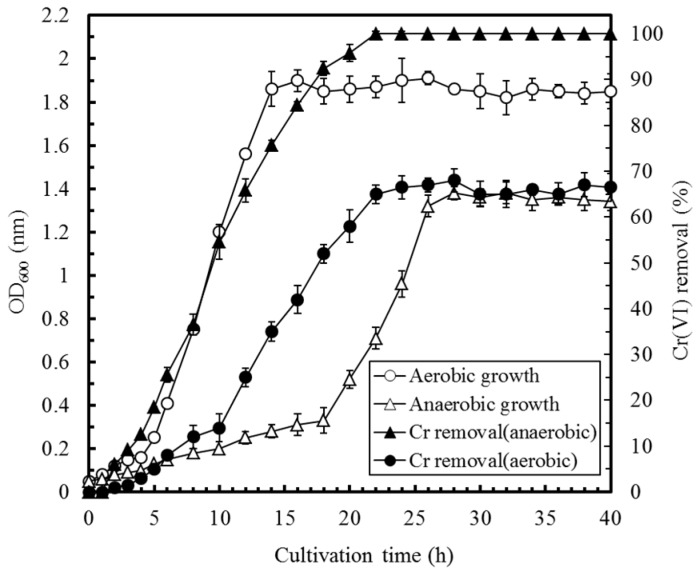
Effects of cultivation time on the cell growth of *E. aestuarii* YC211 and Cr(VI) removal efficiency in TSBCr medium with 60 mg/L Cr(VI) under aerobic and anaerobic conditions at 30 °C, pH 7.0, and 40 h of incubation (error bars represent standard error; values are presented as the mean of triplicate measurements).

Figure 2 delineates the effects of cultivation time on the cell growth and Cr(VI) removal by *E. aestuarii* YC211 under aerobic and anaerobic conditions. The results indicated that the logarithmic growth phase of *E. aestuarii* YC211 ranged from 4 h to 14 h and from 18 h to 26 h under aerobic and anaerobic conditions, respectively. The specific growth rates (μ) of *E. aestuarii* YC211 were 0.47 h^−1^ and 0.16 h^−1^ under aerobic and anaerobic conditions, respectively. As for Cr(VI) removal by *E. aestuarii* YC211, the optimal efficiencies were 67.5% (at the 28th h) and 100% (at the 22nd h) under aerobic and anaerobic conditions, respectively. During the logarithmic growth phase, the growth of *E. aestuarii* YC211 and Cr(VI) removal were synchronous under anaerobic conditions but not under aerobic conditions. Although *E. aestuarii* YC211 grew quickly under aerobic conditions, the amount of Cr(VI) transformed per OD_600_ of growth was high under anaerobic conditions. Similar results were reported for Cr(VI) removal by *E. aurantiacum* under various oxygen conditions [27]. Thus, the inoculation time was set at 22 h for the subsequent experiments, which were conducted under anaerobic conditions, unless otherwise stated. As compared with previous studies using *Ochrobactrum anthropi* YC152, *E. aestuarii* CE1, *Exiguobacterium* sp. Chr-43, *E. aurantiacum,* and *E. acetylicum* to remove Cr(VI) under similar conditions [7,27,28,29,30], *E. aestuarii* YC211 exhibited high Cr(VI) removal under anaerobic conditions. Because *E. aestuarii* YC211 would be utilized as an exoelectrogenic bacterium in MFC, anaerobic conditions favored the reduction of Cr(VI) to Cr(III).

### 3.2. Factors Affecting Cr(VI) Removal by E. aestuarii YC211

**Figure 3:**
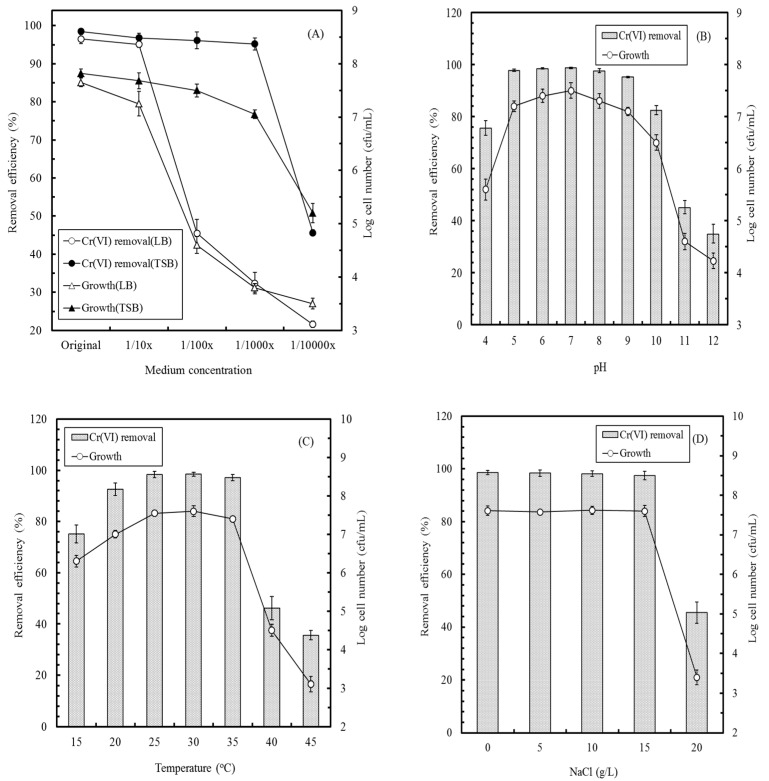
Effects of (**A**) medium concentration (pH:7, temperature: 30 °C, NaCl concentration: 0 M); (**B**) pH (temperature: 30 °C, NaCl concentration: 0 M); (**C**) temperature (pH:7, NaCl concentration: 0 M); and (**D**) NaCl concentration (temperature: 30 °C, pH:7) on the cell growth and Cr(VI) removal efficiency of *E. aestuarii* YC211 (Cr(VI) concentration was: 60 mg/L).

Because *E. aestuarii* YC211 is inoculated in the anode of the MFC to simultaneously remove Cr(VI) and generate electricity, the factors affecting Cr(VI) removal by *E. aestuarii* YC211 were evaluated. Figure 3A illustrates the effects of different kinds of media and their concentrations on the cell growth and Cr(VI) removal efficiency of *E. aestuarii* YC211. The results indicated that TSB was more suitable than LB for *E. aestuarii* YC211 both for Cr(VI) removal and bacterial growth. A nonsignificant effect of the TSB concentration on the Cr(VI) removal efficiency of *E. aestuarii* YC211 was observed when it was in the range of original to 1/1000 TSB. Thus, 1/1000 TSB was used in the subsequent experiments, based on cost considerations. As the pH of the wastewater containing Cr(VI) is variable, the effect of pH on the Cr(VI) removal efficiency of *E. aestuarii* YC211 was evaluated. It can be seen in Figure 3B that a pH of 4–10 led to >75% removal efficiency; however, at an optimal pH range of pH 5–9, >95% removal efficiency could be achieved. This characteristic can favor the operation of the MFC biosensor for measuring Cr(VI) concentration in different water bodies.

Figure 3C illustrates the effects of temperature on the cell growth and the Cr(VI) removal efficiency of *E. aestuarii* YC211. The results indicated that the effects of temperature on the cell growth and Cr(VI) removal efficiency were similar. Relatively high Cr(VI) removal efficiencies were obtained in the range of 20–35 °C, with the highest removal efficiency of 98.5 ± 0.8% being observed at 30 °C. At 15 °C and 40 °C, removal efficiencies of >75.2 ± 3.1% and >46.2 ± 4.5%, respectively, could be achieved. Previously, it was reported that NaCl exerts a negative effect on the activity of microbes, and consequently, on the MFC performance [13,31] Thus, the effect of NaCl concentration on the cell growth and Cr(VI) removal efficiency of *E. aestuarii* YC211 was investigated. Figure 3D indicates that the Cr(VI) removal efficiency of *E. aestuarii* YC211 was not significantly affected by increasing NaCl concentrations (0–15 g/L); the removal efficiencies were in the range of 97.5 ± 1.3% to 98.6 ± 0.8%. A removal efficiency of 45.2 ± 0.3% was achieved when the NaCl concentration was 20% (approximate concentration in seawater). These results suggest that the MFC biosensor inoculated with *E. aestuarii* YC211 can be potentially used to measure Cr(VI) concentrations at various salt concentrations. Such characteristics of *E. aestuarii* YC211 indicate that it is more environmentally than adaptable *E. acetylicum*, *O. anthropi* YC152, and *E. aestuarii* CE1 [7,28,30]. The mesophilic, alkalotolerant, and extreme halotolerant characteristics of *E. aestuarii* YC211 suggest that the MFC biosensor inoculated with the YC211 strain can be used for measuring Cr(VI) concentrations in different water bodies.

### 3.3. Effect of Coexisting Ions on the MFC Performance

**Figure 4:**
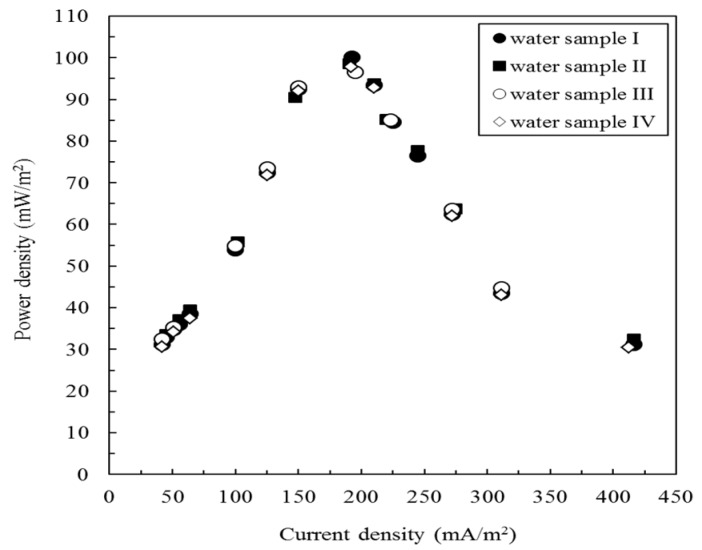
Power curves obtained from the microbial fuel cell (MFC) biosensor inoculated with *E. aestuarii* YC211 during the stable phase of power generation under different water qualities (operating temperature: 30 °C, anolyte: water I to IV, catholyte: 100 mM PBS and 100 mM NaCl).

Actual wastewater contains several types of ions and the presence of these ions in the water samples may affect the activity of the microbes in the anode of the MFC or interferes with the electron transfer capability of the ion-exchange membrane in the MFC, which may reduce the performance of the MFC [32]. Thus, it is important to evaluate the effect of the coexisting ions in the water samples on the performance of the MFC. In this study, 1/1000 TSB (water sample I) and three other water samples (water sample II–IV) containing different cations or/and anions were tested. The potential output from the MFC biosensor was evaluated by gradually increasing the circuit load, ranging from 100–10,000 Ω. The results indicated that the MFC potential increased with increasing resistance, which is typical of a typical MFC system. As shown in Figure 4, the power density initially increased with an increase in the current density but started to decrease after a certain point. The performance of the MFC was not significantly affected by water quality (*p* > 0.05). The maximum power densities for different water samples ranged from 96.5 mW/m^2^ ± 2.8 mW/m^2^ to 100.1 mW/m^2^ ± 1.2 mW/m^2^. These results illustrate that *E. aestuarii* YC211 is an exoelectrogenic bacterium with high environmental adaptability; however, its electron transfer mechanisms are not clear at present. The average maximum power density of the MFC biosensor was 98.3 mW/m^2^ ± 1.5 mW/m^2^ at 1500 Ω. Thus, the external resistance was set at 1500 Ω for subsequent experiments. The maximum power density of the MFC using *E. aestuarii* YC211 was greater than that of the Cr(VI)-MFC biosensor using *O. anthropi* YC152 (89.1 mW/m^2^ ± 1.2 mW/m^2^) [7]; thus, the MFC using *E. aestuarii* YC211 may exhibit high signal strength and thus further widen the Cr(VI) concentration measurement range.

### 3.4. Effects of Cr(VI) Concentration on the Voltage Output of the MFC

**Figure 5:**
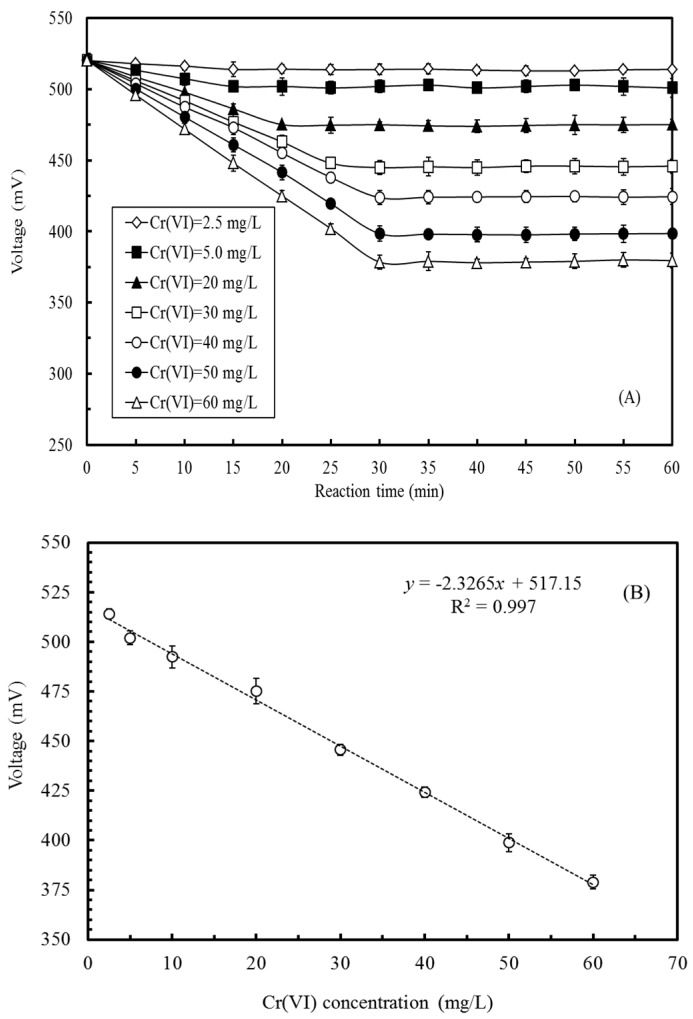
(**A**) Profile of voltage output of the MFC biosensor inoculated with *E. aestuarii* YC211 under different Cr(VI) concentrations; (**B**) Relationship between Cr(VI) concentration and voltage output of the MFC biosensor inoculated with *E. aestuarii* YC211 (operational temperature: 30 °C, resistance of external circuit: 1500 Ω, anolyte: 1/1000 TSB supplemented with different Cr(VI) concentrations, catholyte: 100 mM PBS and 100 mM NaCl).

To establish the calibration curve between the MFC voltage and Cr(VI) concentration in the MFC, 1/1000 TSB, supplemented with different Cr(VI) final concentrations (0.01–100 mg/L), was introduced into the anode compartment of the MFC in a batch mode. In the anode, the inoculated *E. aestuarii* YC211 oxidized the organic compounds and reduced Cr(VI) which acted as the electron acceptor in the anode. The higher the Cr(VI) concentration in the anode, the fewer were the electrons transferred to the cathode via external resistance. Thus, the MFC voltage was expected to decrease with increasing Cr(VI) concentration. Figure 5A indicates that the MFC voltage decreased with reaction time and gradually reached a stable value. Higher Cr(VI) concentrations required longer reaction times to achieve a stable voltage output. Reaction times of 15 min, 25 min, and 30 min were required for stable voltage production when the Cr(VI) concentrations were 2.5–25 mg/L, >25–45 mg/L, and >45–60 mg/L, respectively. However, while a nonsignificant change was observed in the voltage output of the MFC at a relatively low Cr(VI) concentrations, the change was dramatic at high Cr(VI) concentrations. When compared with the condition in which the Cr(VI) anolyte was absent, the anolytes with 5 mg/L, 30 mg/L, and 60 mg/L of Cr(VI) reduced the MFC voltage by 3.6%, 14.3%, and 27.3%, respectively. Although the sensitivity of the Cr(VI)-MFC biosensor was relatively low compared with that of in the cube MFC developed by Liu et al. (2014), the flat microliter membrane-based MFC developed by Xu et al. (2015), and the Cr(VI)-MFC biosensor inoculated with *O. anthropi* YC152 by Wang et al. (2016), the Cr(VI)-MFC biosensor could measure Cr(VI) concentrations across a broader ranges [7,12,19]. Per the results shown in Figure 5A, a reaction time of 30 min was required to obtain a stable voltage output.

Figure 5B illustrates the relationship between the MFC voltage and the Cr(VI) concentration in the MFC in a batch mode. A strong linear relationship could be observed when the Cr(VI) concentration was in the range of 2.5 mg/L to 60 mg/L. The regression equation for the relationship between MFC voltage and the Cr(VI) concentration was determined to be *y* = −2.3265*x* + 517.15 (*r*^2^ = 0.997). When the Cr(VI) concentration was <2.5 mg/L, the extent of voltage decrease was too small to establish a relationship between the MFC voltage and the Cr(VI) concentration in the MFC. When the Cr(VI) concentration was >60 mg/L, the voltage variation was too dramatic to evaluate their relationship; this phenomenon may be attributed to the toxicity of concentrated Cr(VI) to *E. aestuarii* YC211. Using the calibration curve, the Cr(VI) concentration in the wastewater can be rapidly determined. Most regulatory agencies state that the maximum allowable level of Cr(VI) in effluents is 0.5 mg/L; however, the concentrations of Cr(VI) emitted in the processes should be strictly monitored in order to ensure green and clean production. Thus, the developed Cr(VI)-MFC biosensor, which has a measurement range of 2.5–60 mg/L, is suitable for in situ Cr(VI) measurement, especially for the effluents produced by different electroplating units.

It is desirable that the MFC biosensor should exhibit a stable performance during operation. To evaluate this characteristic, 1/1000 TSB, containing different concentrations of Cr(VI) (5, 25, or 50 mg/L), was introduced into the MFC to understand the voltage changes in the MFC biosensor in a batch mode. Approximately 108 h were required for the MFC biosensor from the moment that it started to reach the maximum voltage, and then for the voltage to gradually reduce to 1/10th of its maximum value. When the MFC voltage was 1/10th of the maximum voltage, two-thirds of the anolyte was replaced with the fresh medium. The results of 20 similar cycles for different Cr(VI) concentrations demonstrated the operational stability of the MFC biosensor. The reproducibility of the MFC biosensor in 20 cycles was 2.7%, 6.0%, and 3.6% for 5, 25, and 50 mg/L Cr(VI) concentrations, respectively. Theoretically, when the anolyte in the MFC was periodically refreshed, the MFC performance could be maintained intact over long periods of time.

### 3.5. Cr(VI) Measurement in Artificial and Actual Electroplating Wastewater

Table 1 summarizes the measured Cr(VI) concentrations in the artificial wastewater and electroplating wastewater that was obtained from various operational units; the concentrations are measured using the MFC biosensor or through the standard colorimetric method. The results indicated deviations in the range of −8.7% to 8.0% in the Cr(VI) concentrations measured by the MFC biosensor; on the other hand, the results obtained with the standard colorimetric method exhibited deviations in the range of −3.6% to 3.4%. It can be inferred that standard colorimetric method was slightly more accurate than using the MFC biosensor. Moreover, when the values determined using the MFC biosensor and the standard colorimetric method were compared, <10% deviation (−9.3% to 9.5%) was observed at all the tested concentrations. A strong linear relationship (*y* = 1.042*x* − 0.9848, *r*^2^ = 0.992) could be found between the values determined by the MFC biosensor and the standard colorimetric method. These results demonstrate that the MFC biosensor is about as accurate as the standard method. Thus, the MFC biosensor can be potentially used to determine Cr(VI) concentration in wastewater. Table 1 also lists the Cr(VI) concentrations in the actual electroplating wastewater from different electroplating units, measured using either the MFC biosensor or by colorimetric method. The biosensor-based measurements on actual wastewater were relatively accurate and exhibited low deviations (−6.1% to 2.2%) as compared with the results that were obtained with colorimetric method. Thus, the results indicate that the Cr(VI)-MFC biosensor can be used for accurate in situ measurement of Cr(VI) concentration in electroplating wastewater.

**Figure 6:**
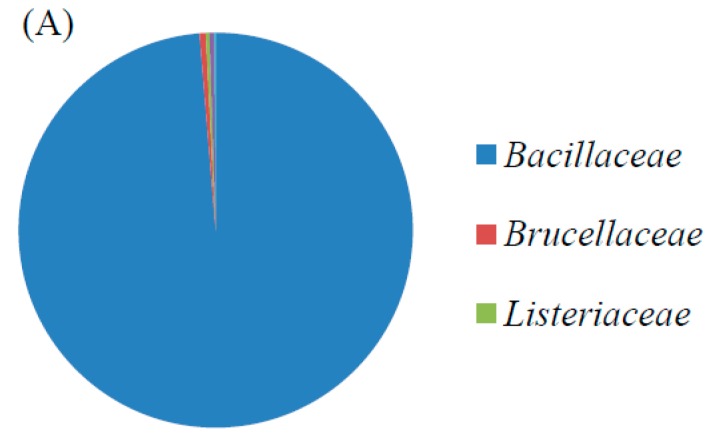

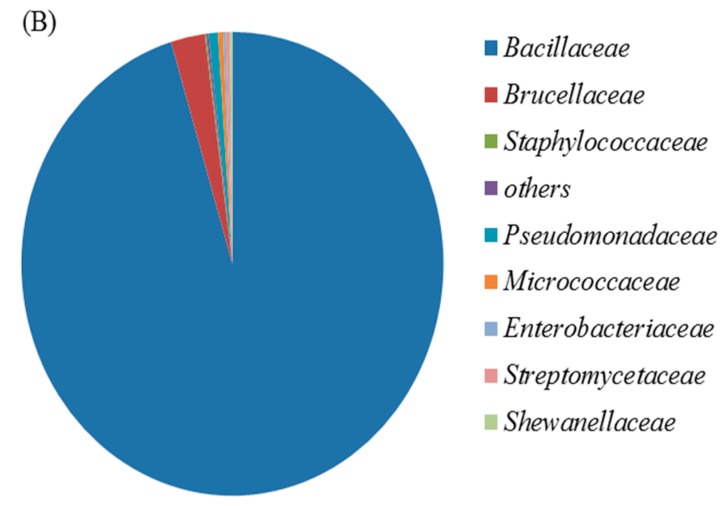
Relative abundances of the bacterial 16S rRNA gene sequences at the family level in the biofilm of MFC (**A**) before; (**B**) after treating actual electroplating wastewater.

To understand the changes in the bacterial community of the MFC before and after operation, the biofilm at the anode was analyzed using NGS. Figure 6A,B indicate the relative abundances of the bacterial 16S rRNA gene sequences at the family level in the biofilm of the MFC before and after treating real electroplating wastewater, respectively. When the biofilm in the MFC was completely immobilized or before the actual electroplating wastewater was treated, the most abundant families that were found in the biofilm were *Bacillaceae* (98.7%), *Brucellaceae* (0.5%), *Staphylococcaceae* (0.4%), and *Listeriaceae* (0.3%). Together, these families accounted for 99.9% of the total bacterial sequences obtained. After the MFC was used to treat the actual electroplating wastewater, the most abundant families found in the biofilm were *Bacillaceae* (95.3%), *Brucellaceae* (2.6%), and *Pseudomonadaceae* (0.7%). Together, these families accounted for 98.6% of the total bacterial sequences obtained. Other families included *Micrococcaceae* (0.4%), *Streptomycetaceae* (0.3%), *Enterobacteriaceae* (0.2%), *Shewanellaceae* (0.2%), and *Staphylococcaceae* (0.1%). The bacterial community in the MFC, after it was used to treat actual electroplating wastewater, was more abundant than that in the original MFC but the dominant families were similar (i.e., *Bacillaceae* and *Brucellaceae*). Joutey et al. (2015) reported that chromate-reducing bacteria include *Arthrobacter* sp., *Bacillus* sp., *Ochrobactrum* sp., *Providencia* sp., *Pseudomonas* sp. and *Streptomyces griseus* [33]. *Shewanella oneidensis* MR-1 has been used as a biocathode in MFCs to reduce Cr(VI) [34]. Gupta et al. (2012) revealed the ability of *Exiguobacterium* sp. to tolerate or remove Cr(VI) [28]. This clearly explains why *Bacillaceae* and *Brucellaceae* were the dominant families, even after treating real electroplating wastewater with the MFC over a long period of time; inoculated *E. aestuarii* YC211 belongs to the family *Bacillaceae* and the chromate-reducing bacteria, *Ochrobactrum* sp., belongs to the family *Brucellaceae*. *Arthrobacter* sp., *Bacillus* sp., *Providencia* sp., *Pseudomonas* sp., *S. griseus*, and *S. oneidensis* belong to the *Micrococcaceae*, *Bacillaceae*, *Enterobacteriaceae*, *Pseudomonadaceae*, *Streptomycetaceae*, and *Shewanellaceae* families, respectively. The existence of these species in the biofilm was further confirmed by NGS. The aforementioned studies clearly demonstrated that reliable MFC performance might be attributed to a stable bacterial community present during the treatment period.

## 4. Conclusions

In this study, we developed a Cr(VI)-MFC biosensor that was inoculated with *E. aestuarii* YC211 for the in situ determination of Cr(VI) concentration in electroplating wastewater. *E. aestuarii* YC211 is facultatively anaerobic, Cr(VI)-reducing, and exoelectrogenic; further, it has high adaptability to pH, temperature, salinity, and water quality. Our results illustrate that the MFC biosensor is a simple but reliable device for measuring a wide range of Cr(VI) concentrations with high accuracy in a short period of time. By NSG analysis, the predominant strain in the biofilm was found to be *E. aestuarii*, which accounted for 95.3% of the total bacterial sequences in the MFC even after treating actual electroplating wastewater. This gives us to understand that the reliable performance of the MFC may be attributed to the stable bacterial community present in the MFC during the treatment period. Although the limit of detection of the developed MFC biosensor was slightly larger than the maximum allowable level of Cr(VI) in an effluent, the biosensor was not designed to evaluate whether the Cr(VI) concentration met with wastewater discharge standards or not, instead of being early warming function. Thus, the application of MFC biosensors as in situ devices for Cr(VI) determination in the effluents produced from different operational units in an electroplating plant is promising.

## Figures and Tables

**Table 1:** Cr(VI) measurement from artificial wastewater and actual wastewater by MFC biosensor and colorimetric method.

	Artificial Wastewater						Electroplating Wastewater			
	Standard Cr(VI) Concentration of (mg/L)									
	7.5	15	25	35	45	55	A	B	C	D
MFC biosensor	8.1 ± 0.25	13.7 ± 0.67	25.8 ± 1.35	36.3 ± 2.05	47.1 ± 2.80	52.6 ± 1.55	32.5 ± 2.16	50.8 ± 1.81	278 ± 9.43	516 ± 13.52
Colorimetric method	7.4 ± 0.15	15.1 ± 0.75	24.1 ± 1.02	36.2 ± 2.12	46.4 ± 1.81	56.2 ± 2.07	31.8 ± 2.37	53.6 ± 2.62	296 ± 12.27	543 ± 15.18
Deviation (%) ^1^	8.0	−8.7	3.2	3.7	4.7	−4.4	–	–	–	–
Deviation (%) ^2^	−1.3	0.7	−3.6	3.4	3.1	2.2	–	–	–	–
Deviation (%) ^3^	9.5	−9.3	7.1	0.3	1.5	−6.4	2.2	−5.2	−6.1	−5.0

^1^ The determined value by MFC biosensor compared to standard Cr(VI) concentration; ^2^ The determined value by colorimetric method compared to standard Cr(VI) concentration; ^3^ The determined value by MFC biosensor compared to that by colorimetric method.
